# Can transfer type and implant angulation affect impression accuracy? A 3D in vitro evaluation

**DOI:** 10.1007/s10266-021-00619-y

**Published:** 2021-06-01

**Authors:** Davide Farronato, Pietro Mario Pasini, Veronica Campana, Diego Lops, Lorenzo Azzi, Mattia Manfredini

**Affiliations:** 1 Department of Medicine and Surgery, School of Dentistry, University of Insubria, Varese, Italy; 2 Department of Medicine and Surgery, School of Dentistry, University of Insubria, Varese, Italy; 3 Private Practitioner, Varese, Italy; 4 Department of Prosthodontics, School of Dentistry, University of Milan, Milano, Italy; 5 Department of Medicine and Surgery, Unit of Oral Pathology, Dental Clinic, University of Insubria, ASST Dei Sette Laghi, 21100 Varese, Italy; 6 Private Practitioner, Milano, Italy

**Keywords:** Dental implants, Impression coping, Implant impression technique, Impression accuracy, Implant angulation

## Abstract

Impression accuracy is fundamental to achieve a passive fit between implants and the superstructure. Three transfer types were tested to evaluate the differences in impression accuracy and their efficiency in case of different implant angles. A master model with four implant analogues placed at 0°, 15° and 35° was used. 27 impressions were taken with three different types of impression coping: closed tray technique coping (CT), open tray technique coping (COT) and telescopic open tray coping (TOT). The impressions were poured. Analogues were matched with scan bodies to be scanned and exported in STL. An implant bar was designed from each STL and another one from the master model. A comparison between these bars was obtained. Linear and angular measurements for every type of coping were calculated for different angulations. The collected data were analyzed with ANOVA test (95% of confidence). Student’s *t* test showed a significative discrepancy (*p* ≤ 0.001) on linear and angular measurements on Δ*x*, Δ*y*, Δ*z* with different transfer types as well as diverse implant positioning angles (*p* ≤ 0.001). Within the limitations of this study, it can be concluded that the coping type and the implants divergence may be significant parameters influencing the impression accuracy.

## Introduction

The current state-of-the-art reported in literature highlights how a pivotal role is played by a passive fit of the superstructure on implants for a long-term prosthetic outcome [1–4]. To gain a passive fit of the structures, one of the crucial challenges is an accurate impression taking. Representing one of the first steps in prosthetic rehabilitation, a lack of accuracy during impressions leads to imprecisions to the further steps, such as the technical laboratory phases. The final result is frequently an absence of precision and a misfit of prostheses.

Nowadays it is known how superstructure misfit may result in strain and distortion of its parts, with several complications in both biological and mechanical aspects of the rehabilitation. Concerning the mechanical ones, the most commonly reported are screw loosening, fracture of the prosthetic or implant components [5–12] and prosthetic mainframe damage [12–15]. Among the biological complications, instead, loss of osseointegration [16] should be considered. Indeed, one of the major differences between osseo-integrated implants and natural teeth is represented by the absence of periodontal ligament. While in prosthodontics over natural teeth, the periodontal ligament represents an important element to compensate for the lack of passivity or inaccuracy, when implants are involved its absence, associated with a prosthetic misfit, can lead to loss of osseointegration [17]. For the aforementioned reasons, a precise recording of implants positions is fundamental to obtain properly supported definitive restorations and reduce the stress to implants and the prosthetic framework.

Impression accuracy may be influenced by several aspects. Many researches are aimed to study these factors affecting the precision of impression procedures over implants. The main variables are represented by different impression techniques (direct or indirect), use of various impression materials and relative implant angulation [14]. Depending on the type of transfer used, open-tray or closed-tray impression technique could be used. The literature agreed that different results of implant accuracy are achieved depending on the employed impression technique [18–23]. Most of the study report that the open-tray technique is more precise and predictable than the closed-tray technique [14, 19–23].

Regarding impression materials, polyether and polyvinyl siloxane are the most frequently used for impression recording in implant prosthodontics. Nevertheless, many studies reported that no particular advantages seem to be achieved using one material or the other, addressing both of them to be suitable [19].

Concerning the third element affecting impression accuracy, a mention is due to the use of angulated implants [14, 23, 24]. So far many studies were carried out with parallel implants [25–29], and only a few were focused on nonparallel implants [30–32]. These in vitro studies aimed to research how to improve impression accuracy, but in ideal conditions, frequently very different from the real situations that the clinician must face in daily practice.

In this study, to implement the current state-of-the-art, implants were placed with different angulations in the master model. The absence of parallelism allows to assess the efficiency of impression copings in situations more similar to the clinical reality.

Therefore, the present study aimed to compare the three-dimensional accuracy of three kinds of impression coping produced by a single company: a closed-tray (CT), an open-tray (COT) and a telescopic open-tray (TOT) transfers. At the same time, it is tested their efficiency when used with implants placed with different relative angles.

## Materials and methods

### Fabrication of master model

An acrylic resin rectangular block, namely model A, was produced with a iPro 8000 SLA 3D printer (3D System, Rock Hill, Carolina del Sud, U.S.A.). The STL file of model A was designed (SolidWorks Software, Dassault Systèmes SolidWorks Corporation, Waltham, MA) to fit four holes, with 10 mm of distance between each, to lodge four implant replicas (V2 AL, Kristal Srl, Trezzano sul Naviglio, Italy), with different inclinations. Holes presented a diameter of 4 mm. The hole directions were digitally set as described: the first one, hole for the control analogue, was set at 90° inclination, referred to the horizontal plane of the block. The second, third and fourth hole inclinations were set at 0°, 15° and 35°, respectively, referring to the first hole. Analogues (V2 AL, Kristal Srl, Trezzano sul Naviglio, Italy) were inserted into the holes of the model and cemented in place using an auto-polymerizing acrylic resin (DuraLay; Reliance Dental Manufacturing LLC, Alsip IL, United States). The analogue connection was positioned 0.5 mm above the surface of the resin block to avoid marginal undercuts, which could increase impression deformations. As a result, four analogues fixed in an acrylic block were obtained: the analogue angular position, referring to the first one (control analogue, C0), was set to 0° for the second (T0), 15° for the third (T15) and 35° for the fourth (T35). Impression trays and model A presented corresponding notches to guide a univocal fitting. On the longer side, notches had a diameter of 5 mm and deepen by 3.50 mm. On the shorter side, notches measure 5 mm in width and deepen by 3.50 mm.

### Impressions set-up

**Fig. 1:**
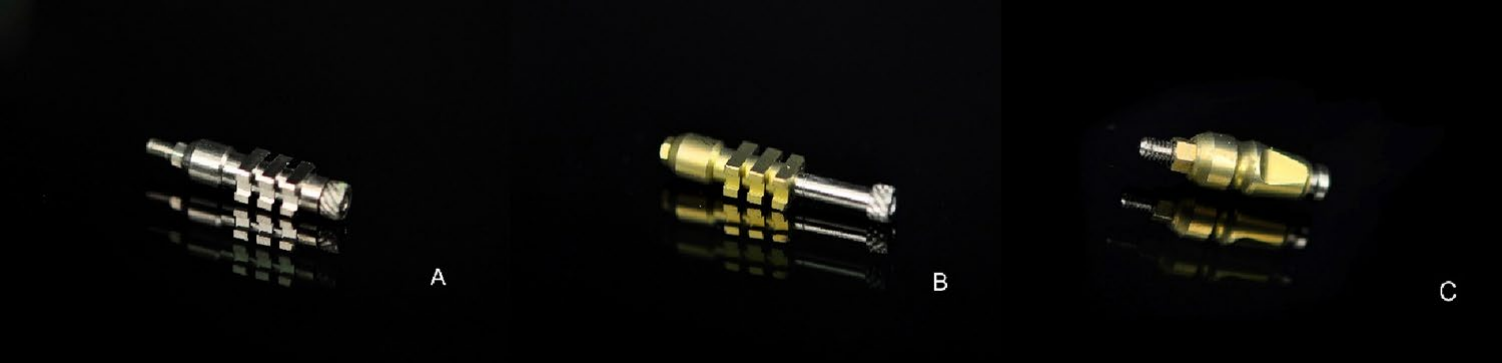
The three transfers under analysis: TOT **A**, COT **B** and CT **C**, respectively

Three types of impression coping were used: closed-tray (CT), classic open-tray (COT) and telescopic open-tray (TOT) (Fig. 1). TOT transfer was characterized by an inner hexagon that can sweep inside the outer body to eliminate any extension of the connection under the conical interface. The length of the mating between the analogue and CT is 1.5 mm, 0.75 mm for COT and 1.5 mm for TOT. All these copings fit on a common implant connection characterized by a 35° conical internal hexed interface (Core, Kristal Srl, Trezzano sul Naviglio, Italy). The implant hex measures 2.42 mm. Three impressions were planned for each combination of transfer (CT, COT, TOT)-analogue inclination (C0, TO, T15, T35), as shown in Table 1. For each impression, only two transfers at a time were connected, not to generate interferences during the investigation of each combination. And for each combination, the impression was repeated three times to ensure that the procedure was carried out correctly. In fact, during the superimposition process, the sample was considered valid only if three impressions presented similar linear and angular deviation values. Instead, if one of them had clearly different values (> 2° and > 1.5 mm), the sample was excluded and the process repeated. Other exclusion criteria were related to the quality of the impressions as described below. The considered variables were three kinds of transfer with three different angulations. The impression procedure was repeated three times for each kind of impression coping, to obtain a total of 27 impressions to be analyzed.

**Table 1:** Impression distribution according to transfer type and implant inclination; the total amount of impressions are 27

	CT	COT	TOT
C0-T0	3	3	3
C0-T15	3	3	3
C0-T35	3	3	3

### Fabrication of custom trays

**Fig. 2:**
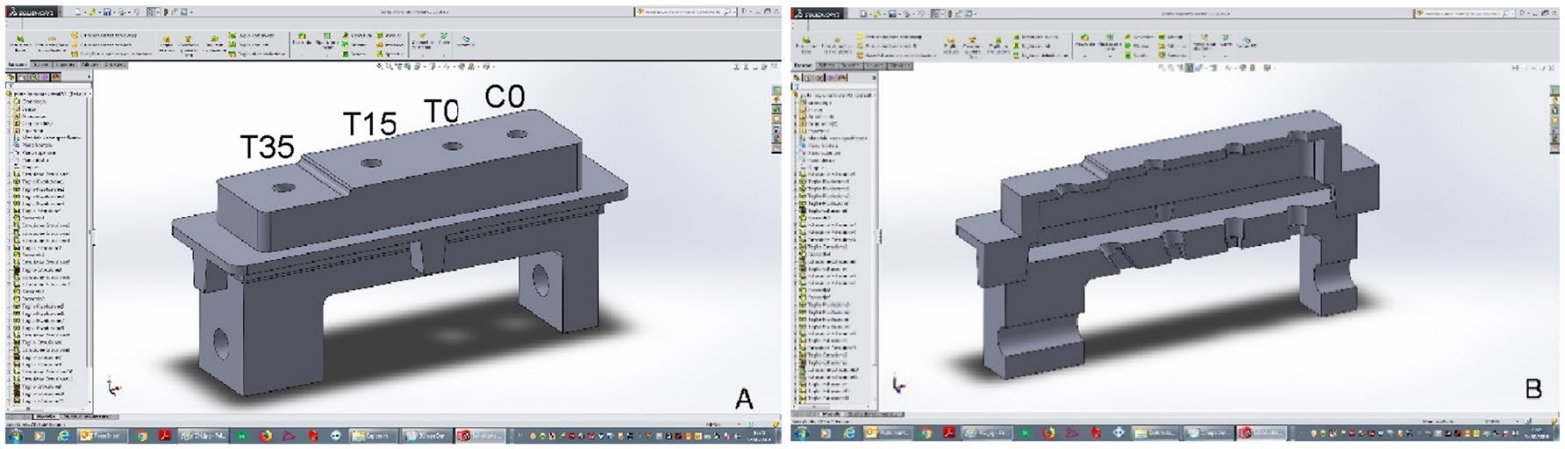
**A** The digital project of the master model and of the individual impression tray. **B** A section of the two structures showing their matching and the holes direction

An acrylic resin (VisiJet M3 Stoneplast; 3D System, Rock Hill, Carolina del Sud, U.S.A) was used to fabricate 27 individual rectangular impression trays with the ProJet MJP 3510 3D printer (3D System, Rock Hill, Carolina del Sud, U.S.A). The individual impression trays and model A were designed with the SolidWorks 3D software (Dassault Systèmes SolidWorks Corporation, Waltham, Massachusetts, United States). The impression trays and the block holding the analogues had corresponding notches, to allow a univocal fitting. On the longer side, notches measure 4.20 mm in width and deepen by 0.90 mm with respect to the inner margin of the tray. On the shorter side, notches measure 5.80 mm in width and deepen by 3.40 mm. Four 4 mm holes were designed in the area of each analogue to grant access to the internal screw according to the open-tray technique. These trays contain the impression material without interfering with the transfer body and the screws (Fig. 2A, B).

### Impression procedures

**Fig. 3:**
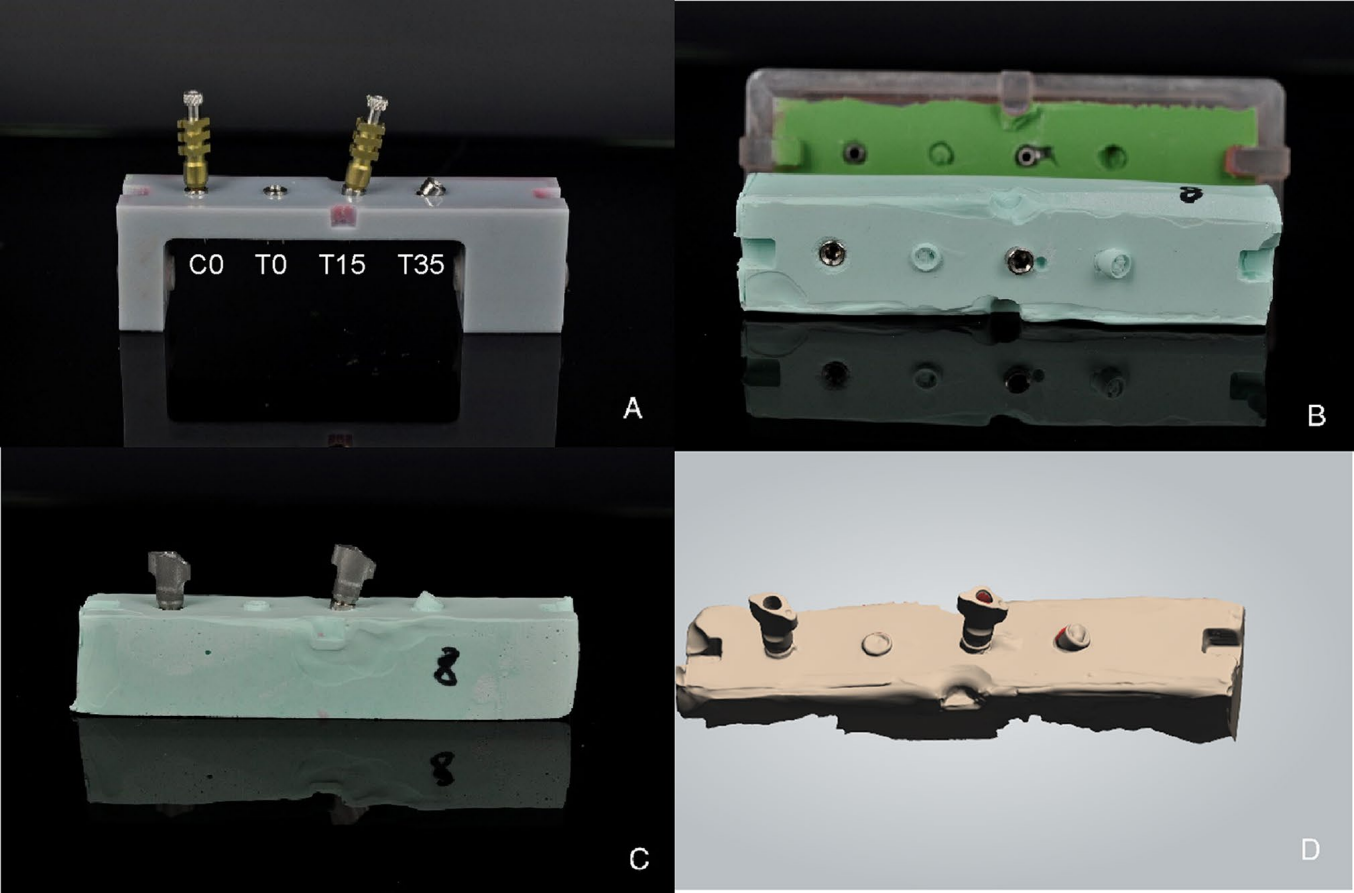
**A** The master model with two sample transfers in place, at 0° (CO) and 15° (T15). **B** An example of impression, with VPS material and its corresponding type IV die stone cast with analogues in place. **C** A cast with scan bodies matched to the analogues under analysis. **D** An STL file revealing the implant’s position referred by the two transfers under analysis

All the impressions were taken by a single operator. Impression phases and materials timing and use described in this article were conducted according to manufacturer instructions. The individual impression trays were brushed with VPS-polyvinyl siloxane adhesive (VPS Tray Adhesive; 3 M ESPE, Seefeld, Germany) 15 min before the impression. Impression copings were fastened on the analogues with 10 Ncm torque. Torque was checked with a dynamometric wrench. No adhesive was brushed on top of the transfers. One-step impression material (Dimension Penta H 3 M, St. Paul, Minnesota, U.S.A.) was chosen for taking the impression of model A. Monophase material was used and prepared with an automated mixing device (Pentamix 2, 3 M ESPE, Seefeld, Germany), using a syringe to bring the material around the transfer copings and performing a direct filling inside the trays. Nine impressions were taken between the two analogues at 0°, C0 and T0, using the three different types of transfer (three impressions each). The same between C0 and T15 (at 15°) and between CO and T35 (at 35°). Each impression was taken on two transfers at a time so that the other transfers did not influence the running evaluation (Fig. 3A). After the impression material hardened, in the open-tray impression, the screws were unscrewed and removed. The individual impression tray was then manually pulled off from the model with a movement as axial as possible referred to C0 axis. Attention was taken to pull the individual impression tray with an axis perpendicular to the base of the model which represented the reference point as it was designed perpendicular to the implant axis. Moreover, during the removing process, the operator was guided by the notches of model A and the corresponding design of the individual impression trays, not to produce moments of force. Implant analogues were connected to the impression transfers, taking care not to impress any rotational movement. The internal screws were screwed using 10 N cm torque. After connecting the analogue, the relationship between polyvinylsiloxane, transfer and analogue was observed at 25 × (Leica Wild M651, Wetzlar, Germany) magnification to detect any gap or distortion. When using a closed-tray impression technique, the holes of the individual impression trays were obturated with a thin layer (approximately 1 mm) of soft wax (Zeta Industria Zingardi Srl, Novi Ligure, Italy) to avoid the dispersion of the impression material. In the closed-tray technique, transfers were linked to the analogues as previously described. The impression was taken and pulled off while the transfers remained attached to the model. The transfers were removed from model A and connected to the implant replicas. The combined transfer-analogue unit was then inserted into the impression, firmly guiding it into place to full depth and slightly rotating it clockwise until an anti-rotational resistance was felt. The presence of bubbles or imperfections in the impression material was set as an exclusion criteria and, if detected, the model was excluded and the impression retaken. Another exclusion criterion was represented by an inconsistent stabilization of the transfer inside the impression due to a lack of material or the detection of a displacement through microscope observation. A total amount of 27 impressions were obtained, consisting of 18 open trays and 9 closed trays. Due to the presence of bubbles and undercuts into the impression material, three impressions (two open trays and one closed tray) were excluded, retaken and substituted. Only impressions that did not present the exclusion criteria listed above were accepted into the study. As indicated by the impression manufacturer (3 M, St. Paul, Minnesota, U.S.A.), at least 30 min was waited before preparing the cast from the impression. Precisely after 60 min from the impression taking, the impressions were boxed with wax and poured with type IV die stone (Zhermack Elite Stone; Zhermack SPA, Badia Polesine, Italy) (Fig. 3B). Plaster manufacturers report a setting expansion after 120 min of 0.08%. After 120 min, the impressions were then removed from stone casts.

### Data acquisition and comparisons

**Fig. 4:**
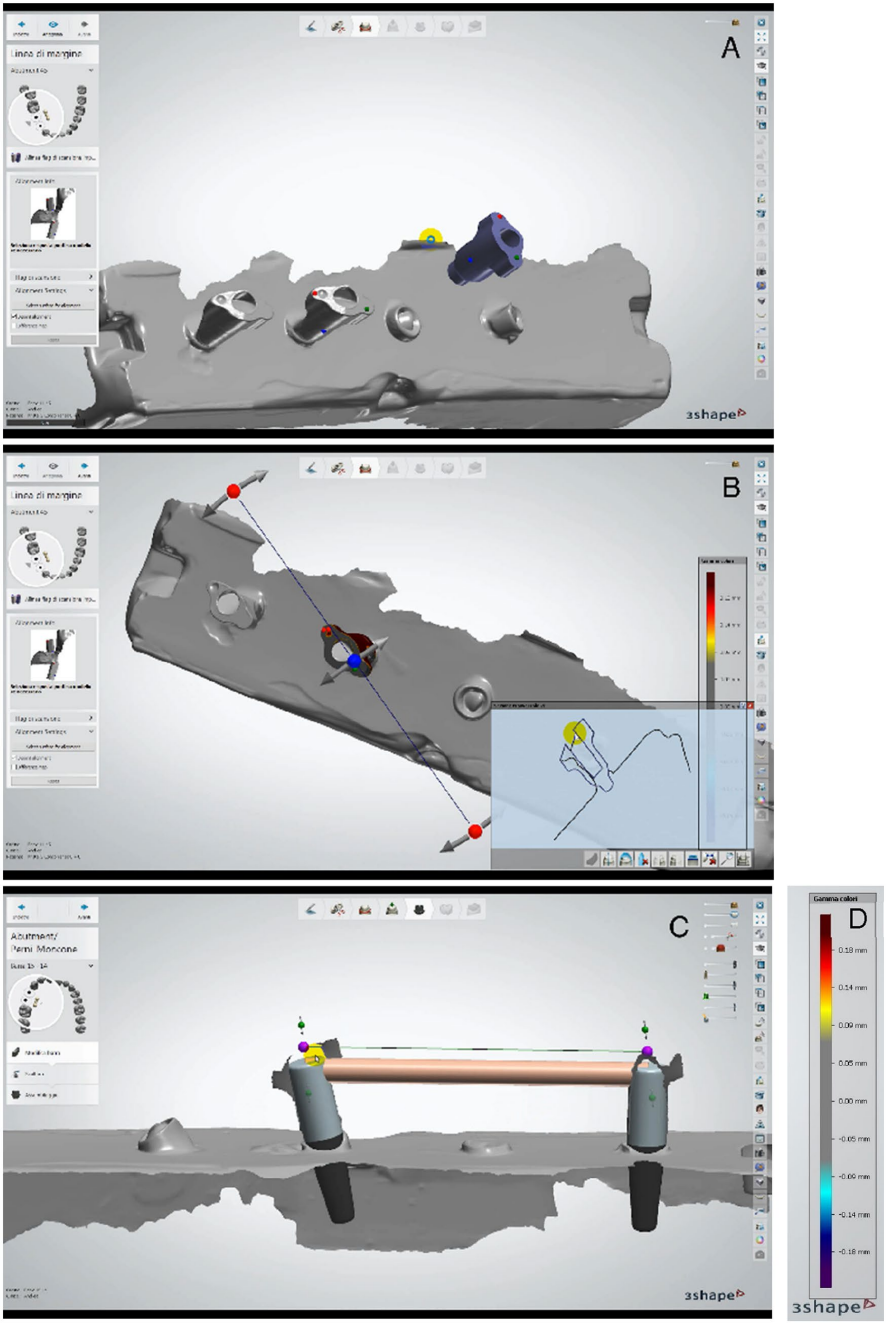
**A** 3 points alignment between the virtual scan bodies and the real one acquired from the cast. **B** A section of the scan body used to verify the matching reliability. **C** A bar projected from the implant’s position acquired. **D** The chromatic scale used to verify the differences in micron during alignment

The master model STL file was produced as described below. Four scan bodies were secured on the four analogues of the master model. The sample was positioned into the industrial scanner 3SHAPE D2000 (documented 5-micron accuracy, ISO 12,836; 4 × 5.0 MP cameras). The acquired data were saved in STL (Standard Tessellation Language) format in a computer file. Using the 3 Shape Dental System software (3Shape, Copenhagen, Denmark) on the STL files, an alignment between the virtual scan bodies and the real scan bodies, acquired from the stone casts, was performed. The real scan bodies were all produced from the digital scan bodies design. A three-point alignment was set between the virtual scan bodies and the real ones, to obtain a match and enhance precision (Fig. 4A). The scan body presented a vestibular notch and two well recognizable parts, one mesial and one distal to the notch. These regions of the scan body were chosen as landmark points for all the digital pairings. To align, three points were selected on the vestibular notch and the mesial and distal part, respectively, on the real scan body and the virtual one. The aligned sections of the two scan bodies were then evaluated to ensure accuracy. The evaluation was obtained with a cross-section of the scan body and with a gradient chromatic scale, a tool of the 3 Shape Dental System software (3Shape, Copenhagen, Denmark), that showed the differences in micron between the virtual and real scan body (Fig. 4B, D). The accepted level of error was set to 40 microns of tolerance between the scan body scansion and the library’s one. An alignment was achieved with the positioning of the three points and the application of a best-fit alignment algorithm that can be verified through a colour map and evaluation sections. The best-fitting between the four scan bodies was not accepted if there was an error above 40 microns when matched. Once the STL cylinders were set, corresponding virtual analogues were generated. Four cylinders were positioned on top of the digital analogues and a bar was designed. This virtual bar (RB) was connected to all four analogues and corresponds to their position as a control reference. All the generated cylinders had equal dimensions in all the samples.

The test samples were produced connecting two scan bodies to the two analogic analogues of the 27 test models, according to Table 1 distribution (Fig. 3C). STL files were acquired for every combination (Fig. 3D). The coupling was performed as described above for the master STL model with accuracy control. STL analogues are therefore positioned. Two cylinders were set on the digital analogue and an implant bar (TB) was designed (Fig. 4C). This procedure was repeated for every pair of transfers, obtaining a total amount of 27 test bars. The bar comparisons were carried out with the industrial software Materialise Magics 13.0, (Materialise, Leuven, Belgium). The STL data of RB and the TB under analysis were imported into the software. The superimposition was obtained by aligning the bar cylinders corresponding to the C0 implant. The concentricity towards the origin of the axes was obtained by placing the Y-axis equal to zero and making an alignment between the two bars, setting as a constraint that the circumferences of the two zero-degree cylinders must be concentric to the origin of the axes. A rotation, on the Z-axis, of the TB on the RB was performed.

**Fig. 5:**
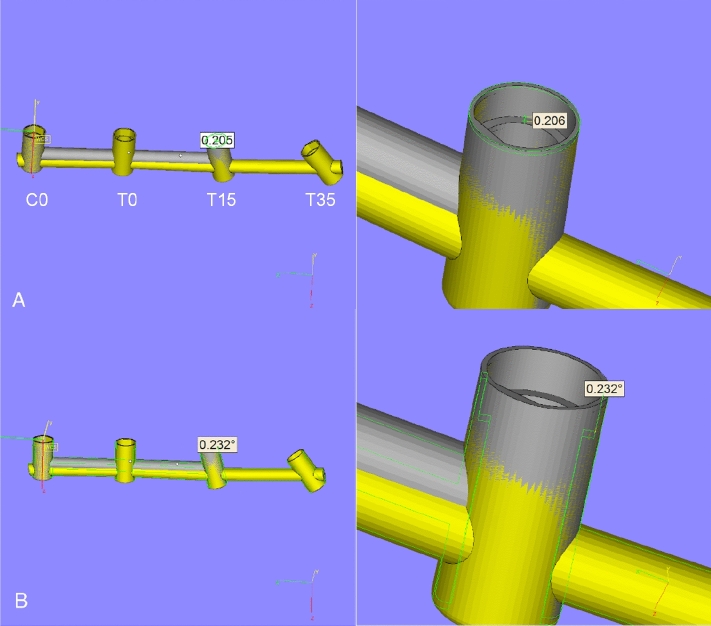
**A** Measurement of the linear and angular displacement of TB compared to RB in X-, Y- and Z-axes. The linear one is lead considering the distance between the center of the outermost circumference of RB and TB’s cylinders. **B** The angular one is obtained from the distance, on plane x–z, between the axis of the two outermost circumferences (one belonging to the RB and one to the TB)

**Fig. 6:**
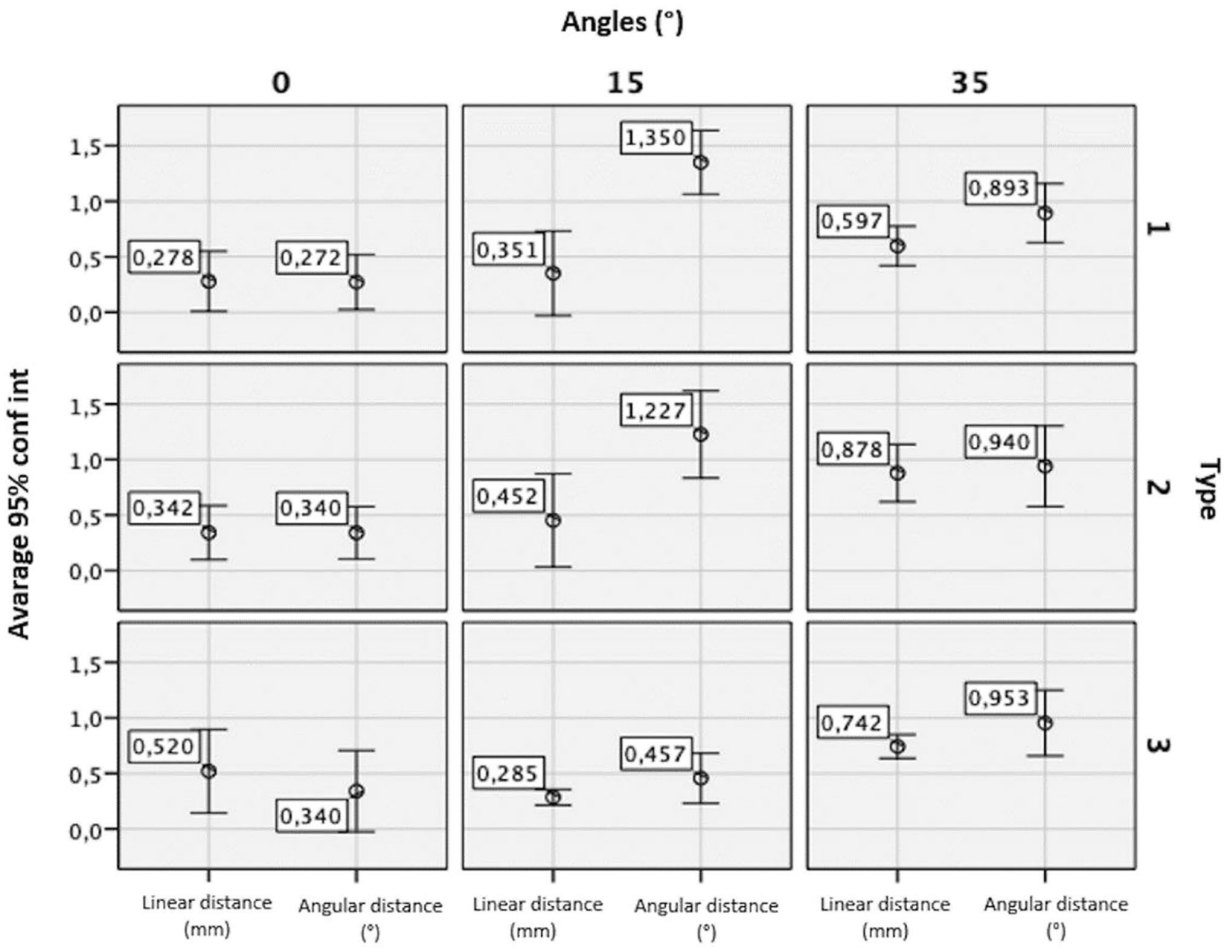
The results of the measurements considering both the transfer types (TOT, COT and CT) and the angle of implant’s position. It is considered a linear and angular displacement

A three-dimensional referent point (REF) was obtained calculating the centre of the coronal circumference of the RB and TB cylinder related to the test analogue. The three-dimensional relative position of the REF points produced a measurement of the linear displacement on the *x*, *y*, and *z* axes (Fig. 5A). The linear 3D measurements on the X, Y and Z coordinates were calculated using the formula [33]: linear displacement = (X2 + Y2 + Z2)1/2 and calculated in millimetres. This process was repeated for all the comparisons. A total amount of 27 linear measurements were obtained. An angular measurement (degrees) was obtained by calculating the distance, on the x–z plane, between the circumference axes of TB, referring to RB corresponding cylinders (Fig. 5B). A total amount of 27 angular measurements were withdrawn (Fig. 6).

One data set belonging to one sample was excluded because one of the three impressions had clearly different values both angular and linear (> 2° and > 1.5 mm). This represented an exclusion criterion, so the sample was not considered valid. Therefore, the impressions and measurements were repeated until the three sets of values were eligible for consideration. A total amount of 27 linear and 27 angular measurements were achieved.

All the collected measurements were processed by the statistical software IBM SPSS Statistics (Armonk, New York, United States). Preliminary statistical analysis was set up running the Shapiro–Wilk test for normality and Levene’s test to assess the equality of variances. A two-way ANOVA test was used for analyzing the relationships between the variables and p-values determined. The level of statistical significance was set at 0.05 and 95% of confidence. The null hypothesis was that there were no different precision levels between the different transfers in use and different angles of analogue placement. It was tested for both linear and angular findings.

## Results

The average differences between TOT, COT, and CT precision levels, within T0, T15 and T35, were shown in Table 2a and b, respectively, for the linear and angular mismatch. When linear measurements were considered, different types of transfer have different precision performances on the mismatch between the REF of RB and TR. TOT shows the greatest precision (0.409 ± 0.289 mm), followed by the CT (0.516 ± 0.281 mm) and the COT (0.557 ± 0.369 mm) demonstrates the least accuracy. When considering the angular measurements, instead, a greater precision was found using the CT (0.583° ± 0.384°), followed by COT (0.836° ± 0.489°) and TOT transfers (0.838° ± 0.523°) (Table 2).

**Table 2:** Average measurements results: linear measurements and st. dev. angular averages and st. dev

Linear averages (mm)	TOT	COT	CT	Overall average
T0	0.278 ± 0.271	0.340 ± 0.243	0,520 ± 0.376	0,380 ± 0,283
T15	0.350 ± 0.380	0.452 ± 0.421	0.285 ± 0.071	0.362 ± 0.295
T35	0.597 ± 0.178	0.878 ± 0.259	0.742 ± 0.107	0.739 ± 0.206
Overall Average	0.409 ± 0.289	0.557 ± 0.369	0.516 ± 0.281	

Angular averages (°)	TOT	COT	CT	Overall average
T0	0.272° ± 0.248°	0.340° ± 0.235°	0.340° ± 0.366°	0.317° ± 0.253°
T15	1.350° ± 0.287°	1.227° ± 0.393°	0.457° ± 0.225°	1.011° ± 0.498°
T35	0.893° ± 0.266°	0.940° ± 0.364°	0.953° ± 0.296°	0.929° ± 0.271°
Overall average	0.838° ± 0.523°	0.836° ± 0.489°	0.583° ± 0.384°	

Regarding the type of transfer in use, a not statistically significant difference on linear (*p* = 0.593) and angular measurements (*p* = 0.429) was detected by the ANOVA test. Regarding the variation of the angle of the transfer, significant values were found in linear (*p* = 0.009) and angular measurements (*p* ≤ 0.001) (Anova).

## Discussion

An accurate impression plays a pivotal role during the prosthetic process, thus the longevity of the rehabilitation [1–3]. The impression precision can be conditioned by various factors, such as the material in use, the technique chosen and the implant angulation. Concerning the first factor, polyvinyl siloxane material (VPS) was selected according to literature evidence that indicates this material as the most suitable for impressions on implants. Parameshwari et al*.* identify polyvinyl siloxane as a material with lower distortion compared to polyether for both non splinted closed and open-tray technique, using custom trays [33]. Sorrentino et al*.* agreed with the previous study [34]. In contrast with these researches, instead, the systematic review presented by H. J. Moreira et al*.* reports more accurate results with the use of polyether (PE) as impression material, followed by VPS [21] in agreement with Sonam Gupta et al*.* [35]. In any case Lee et al*.* [18] and Baig [36], in two systematic reviews, stated that both VPS and polyether represent a valid choice, with no clear advantage between the two. Daoudi et al*.* find no relevant difference between VPS and PE impression materials and affirmed that the choice of impression material makes no significant difference [19]. According to these papers, the material used in the present study seems to be an ideal choice and might decrease as much as possible its influence on the measured outcomes.

Regarding the impression phases, one of the possible bias present in this article can be related to their manual execution. To reduce this aspect all the impression were taken by the same operator. Besides, all the impression trays and the master model presented corresponding notches as points of reference to allow a univocal fitting and guide the operator during the impression procedure (Fig. 2A, B). Another way to reduce this bias could be the use of a mechanical device to take the impression with a standardized and consistent modality, as described by Zarone et al*.* [37] or Piwowarczyk et al*.* [38]. One more modern and digital solution might be represented by the use of augmented reality to guide the tray, always on the same axis and position during the impression protocol [39]. Another possible limitation of the present research are errors that might have been introduced in relation with the evaluation method, in particular, the error when mounting scan bodies and analogues, the scanning one, the error during superimposition and when measuring. All the possible precautions to prevent these errors have been performed, but in any case, it was considered at the statistical analysis a 95% of confidence, or better a 5% of standard error, related to the possible errors in the evaluation method.

About the impression technique, there are two main approaches: the direct one (open-tray) and the indirect one (closed-tray). A study by Patil et al*.* revealed a better accuracy related to the open-tray method rather than to the closed-tray one [20]. A work by Elshenawy et al*.* assumed that the direct technique, above all the splinted one, produces the most accurate casts, followed by the indirect technique [14]. H. J. Moreira et al*.*, in a review of 32 studies, showed that in 14 studies (direct vs indirect) the direct technique (open) was the most accurate in comparison to the indirect one (closed) [21]. Moreira Cabral and Gramani Guedes observed the same findings [22] on a wider sample (*n* = 60). A study by Daoudi et al*.* investigated the main cause of the lower accuracy on 40 stonecasts through the indirect technique and noted that it could be referred to copings repositioning [19]. In their study, the copings difficultly matched with the original position and this was considered to be the most important source of error in the closed-tray technique. Baig and co-workers, instead, stated that conflicting evidence exists regarding the most accurate impression technique (open tray/closed tray) and that no clear recommendation could be given [36]. Rashidan et al*.* also assumed that there is no significant difference between direct and indirect impression techniques [40]. Lee et al. introduced a concept of examined implant number. It was stated that, in situations in which there are ≤ 3 implants, there is no difference between the open-tray and closed-tray techniques, whereas for ≥ 4 implants there is a higher accuracy with the pick-up technique [18]. Besides, Papaspyridakos et al. assessed that the open-tray impression technique was more accurate than the closed-tray impression technique for completely edentulous patients [23]. There seemed to be no difference for partially edentulous patients as well. Again, in this study, no statistical difference in the impression accuracy was found when there were three or fewer implants, anyhow the pick-up technique produced superior accuracy for multiple implants having an angulation of more than 20 degrees. In the present investigation, the amplitude of the techniques was widened to the telescopic transfer (TOT) and the first aim was to compare the precision between TOT, COT and CT. The results of this study suggest that the open-tray technique transfers (telescopic TOT and classic COT) might be the most accurate concerning the linear measurements, in agreement with most of the above-mentioned [14, 18–23] studies. Nevertheless, the sample size was too small. Indeed, statistical significance was not achieved and the null hypothesis could not be confirmed. When the angular measurements were considered, significantly better results were associated with the closed-tray technique (CT). These results did not match with several studies, which state the better accuracy of the open-tray technique. Differently other researches, in agreement with this study, measured that both impression techniques provide comparable results [7, 20]. Again, it must be considered the limitation of the present finding due to the limited number of samples and a wider number should be investigated to confirm the hypothesis. The implant placement angulation is another fundamental factor that may influence the accuracy of the impression [14, 23, 24]. For that reason, the second purpose of this study was to compare the efficiency of different impression types with different implant angulations, to better analyze a clinical situation. Implant angulations of 0°, 15° and 35° degrees were chosen according to the literature attention. Most studies mentioned considered these two angle values as critical thresholds below or above which was possible to notice a change in the precision of the impression [14, 30, 41]. Regarding the angle of implant positioning, a study by Parameshwari et al. two angle values stated that increased angulations tend to decrease the impression accuracy and that the open-tray technique was more accurate with no axially oriented implants (angulation ≥ 15 degrees) [33]. Meanwhile, with parallel implants, there was no statistically significant difference between closed or open-tray technique. Howell et al. two angle values also reported that in case of implant angulation of 30 degrees, the open-tray technique was more accurate than the closed-tray one [41]. Carr and co-workers reported that an angulation under 15 degrees had no effects on impression accuracy [30]. The same author, in an experimental cast with 5 implants, confirmed that with angulated fixtures it could be obtained less accuracy rather than with straight implants [31]. Jang et al. stated that angulations over 20 degrees affected negatively the impression accuracy and that the internal-connection implants were accurate when the angle is ≤ 15 degrees [42]. They also noticed that the more the angle increases, the more the impression accuracy drops and that the inaccuracy was more emphasized for the 20-degree-divergent implants. According to Elshenawy et al. when implant angulation increased from parallel to 30°, the forces of deformation at the impression material were enhanced and this can lead to increased distortions of the impression [14]. Filho et al. also noticed that there were significant angular differences between inclined and straight implants, showing that the less the implant was axially inclined, the greater was the inaccuracy of the impression [43]. Some other authors, though, reported different results: Conrad et al. showed that divergent or convergent implants had no significantly different effect on the accuracy of the definitive casts and that the distortion was similar for different impression techniques, implant angulations, and implant number [44]. In contrast, Al-Abdullah et al. found that the angulation and position of the implants did not have any significant effect on the accuracy of the impression technique [45]. In the present research, angles seemed to play a relevant role in generating impression errors. In fact, statistically significant differences showed T0 scenario as the best solution whereas T15 and T25 compete similarly in a lower precision. As a proof of principle supposition, it might be considered the telescopic impression coping (TOT) as a better solution, compared to COT and even more to CT. The hypothesis was based on the amount of undercut to be overpassed to detach the impression from model A. The bigger the undercut was, the bigger the deformation might be in the silicon material. TOT was characterized by an inner hexagon that could sweep inside the outer body to eliminate any retention of the connection under the conical interface when tilted implants impressions were taken. The retractable solution recorded better performances, especially in T35 situations, both in linear and angular precision. But differently than expected and within the limitation of the sample size, it did not reach any significant difference when compared to the classic CT and COT solutions. One of the reasons might be the introduction of a second tolerance between the inner hexagon and the transfer body as well as the need to plan new studies with wider samples.

## Conclusion

The present study aimed to verify if the impression technique and the implants relative angulation could affect the precision of the impression, thus the longevity of the rehabilitation. The results obtained indicate that implants angulation affects the accuracy of the impression, and therefore the prosthetic result, more than the type of coping used. The greater the relative angle measured between two implants, the more the accuracy was reduced. Furthermore, the impression technique did not produce significant differences in linear and angular mismatches. Further studies and a larger sample are needed in the future.
